# A comprehensive protocol combining in vivo and ex vivo electrophysiological experiments in an arrhythmogenic animal model

**DOI:** 10.1152/ajpheart.00358.2023

**Published:** 2023-11-17

**Authors:** Mathias Nyman, Tomas O. Stølen, Anne Berit Johnsen, Karin Garten, Francis L. Burton, Godfrey L. Smith, Jan Pål Loennechen

**Affiliations:** ^1^Department of Circulation and Medical Imaging, NTNU, Norwegian University of Science and Technology, Trondheim, Norway; ^2^Clinic of Cardiology, St. Olavs University Hospital, Trondheim, Norway; ^3^School of Cardiovascular and Metabolic Health, University of Glasgow, Glasgow, United Kingdom

**Keywords:** defibrillation, ex vivo electrophysiological study, in vivo electrophysiological study, optical mapping, ventricular arrhythmia

## Abstract

Ventricular arrhythmias contribute significantly to cardiovascular mortality, with coronary artery disease as the predominant underlying cause. Understanding the mechanisms of arrhythmogenesis is essential to identify proarrhythmic factors and develop novel approaches for antiarrhythmic prophylaxis and treatment. Animal models are vital in basic research on cardiac arrhythmias, encompassing molecular, cellular, ex vivo whole heart, and in vivo models. Most studies use either in vivo protocols lacking important information on clinical relevance or exclusively ex vivo protocols, thereby missing the opportunity to explore underlying mechanisms. Consequently, interpretation may be difficult due to dissimilarities in animal models, interventions, and individual properties across animals. Moreover, proarrhythmic effects observed in vivo are often not replicated in corresponding ex vivo preparations during mechanistic studies. We have established a protocol to perform both an in vivo and ex vivo electrophysiological characterization in an arrhythmogenic rat model with heart failure following myocardial infarction. The same animal is followed throughout the experiment. In vivo methods involve intracardiac programmed electrical stimulation and external defibrillation to terminate sustained ventricular arrhythmia. Ex vivo methods conducted on the Langendorff-perfused heart include an electrophysiological study with optical mapping of regional action potentials, conduction velocities, and dispersion of electrophysiological properties. By exploring the retention of the in vivo proarrhythmic phenotype ex vivo, we aim to examine whether the subsequent ex vivo detailed measurements are relevant to in vivo pathological behavior. This protocol can enhance greater understanding of cardiac arrhythmias by providing a standardized, yet adaptable model for evaluating arrhythmogenicity or antiarrhythmic interventions in cardiac diseases.

**NEW & NOTEWORTHY** Rodent models are widely used in arrhythmia research. However, most studies do not standardize clinically relevant in vivo and ex vivo techniques to support their conclusions. Here, we present a comprehensive electrophysiological protocol in an arrhythmogenic rat model, connecting in vivo and ex vivo programmed electrical stimulation with optical mapping. By establishing this protocol, we aim to facilitate the adoption of a standardized model for investigating arrhythmias, enhancing research rigor and comparability in this field.

## Graphical abstract

**Figure:**
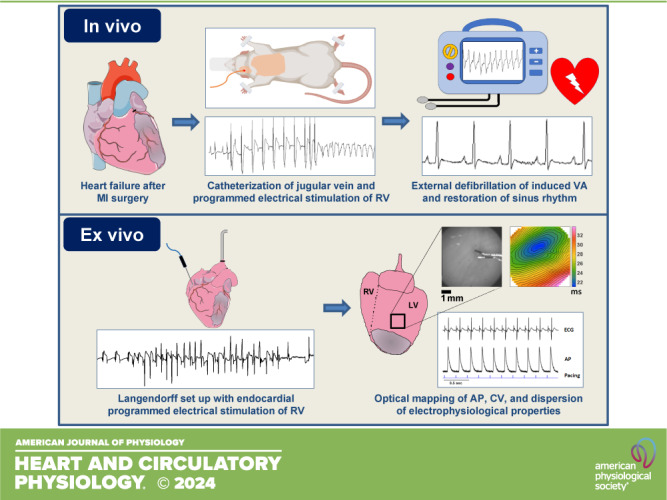

## INTRODUCTION

Cardiovascular disease is the leading cause of mortality, accounting for nearly one in five of total deaths (1). Up to half of all cardiovascular disease-related deaths are attributable to sudden cardiac death caused by ventricular arrhythmia (VA) (2). Myocardial infarction (MI) with heart failure represents the most common cause of life-threatening VA in a clinical setting (3, 4). VAs are mainly triggered by abnormalities in action potential (AP) generation, propagation, or repolarization (5). The dynamics of VA in the context of triggers and substrates are considered multifactorial (6, 7).

Despite advancements in our understanding of single cardiomyocyte electrophysiology (EP), the specific relationship between myocardial disease and arrhythmias at tissue level remains largely unknown. As a result, our current understanding of the combined effect of ion-channel dysfunction and structural changes in cardiac disease is limited, which in turn hinders the development of effective treatment and prevention strategies.

Programmed electrical stimulation (PES) protocols, typically with a multipolar catheter inserted via the femoral vein to the right ventricle, are central in invasive clinical EP studies. Most often, up to three ventricular extrastimuli are applied routinely to induce VA in catheter mapping and ablation procedures, but four extrastimuli can also be used (8). However, these clinical protocols are often not used in experimental studies, preventing translation of the experimental findings to the clinical context and vice versa. Animal models are of major importance to study disease mechanisms and to measure the effects of antiarrhythmic interventions (9, 10). Frequently, EP studies are performed ex vivo in isolated hearts or in vivo in animals (11). In vivo studies are done under more physiological conditions, simulating clinical situations, but give fewer options for mechanistic studies. In comparison, ex vivo studies permit considerable possibilities for more complex protocols and mechanistic measurements.

Life-threatening VAs usually originate in regions of remodeled or scarred tissue due to MI or other myocardial diseases (12, 13). Areas of surviving cardiomyocytes within the scarred zones lead to dispersion of conduction velocity (CV) and refractoriness, mostly by changes in AP waveforms (14). A key aspect to studies on arrhythmogenicity is information on the spatial aspect of repolarization (15). Optical mapping is a widely used technique to study the pathological changes and heterogeneity of cardiac EP (16). This technique enables high spatial and temporal resolution imaging of AP waveforms (17). The steps involved in undertaking in vivo and ex vivo PES in mice have been demonstrated (18). However, it has not been common to perform in vivo EP protocols coupled with ex vivo studies in a disease model, using similar pacing protocols in combination with optical mapping of the AP waveform.

Here, we provide a method for a pipeline of comprehensive multilevel EP analysis in a rat model of heart failure after MI. We have combined several well-established in vivo and ex vivo methods into a series of experiments performed on the same individual.

First, the susceptibility to and characterization of VA were determined by PES in vivo. In case of sustained VA, electrical defibrillation was performed to terminate the VA before commencing the ex vivo experiments. Ex vivo PES was conducted and confirmed the retention of the in vivo phenotype before proceeding with optical mapping experiments. Sequencing the experiment in this order allows us to ensure that the more detailed ex vivo experiments examine the same phenotype observed in vivo.

## ANIMALS

The animals used in this protocol, female Sprague–Dawley rats ∼3 mo old upon arrival, were randomly assigned to either sham surgery or MI surgery. Screening with echocardiography was performed the for assessment of left ventricular ejection fraction. At the time of the in vivo PES, the animals were approximately 6 mo old with a mean weight of 359 g [standard deviation 20.8, confidence interval (350.0, 367.6)]. They were habituated in the animal care facility, were under daily supervision, kept in a 12-h:12-h light/dark cycle, and provided with standard rat pellets and tap water ad libitum. All invasive procedures, including surgery, echocardiography, and in vivo PES, were performed under the general anesthesia outlined in *step B.3*.

The national animal research authority under the Norwegian Food Safety Authority approved the study, which was in accordance with *Guide for the Care and Use of Laboratory Animals* (National Institutes of Health Publication No. 85-23, Revised 1996).

## MI SURGERY

The ligation technique of the left coronary artery is a well-established method (19). In brief, the rats are anesthetized, intubated, and ventilated. Following left thoracotomy, the artery is ligated using a polyester suture to induce MI. During surgery, all animals were administered 1 mg/kg meloxicam (Metacam vet, Boehringer Ingelheim Vetmedica GmbH; Ingelheim am Rhein, Germany) via subcutaneous injection. Meloxicam (1 mg/kg) and buprenorphine (0.1 mg/kg) (Temgesic, Reckitt Benckiser; Hull, UK) were administered approximately 8 and 24 h after the surgery, respectively.

## IN VIVO PROCEDURES

### A) Setting up the Acquisition Hardware and Software System (Timing 60–120 min)

The software and hardware necessary to perform PES should be set up and tested beforehand. In this protocol, we use IOX2 (v. 2.4.2.6), a computer-based data acquisition software (EMKA Technologies, Paris, France) for recording the surface and intracardiac ECG signals. Atrial and ventricular pacing are delivered by an external stimulator generator (STG3000 Series, Multi Channel Systems MCS, Reutlingen, Germany), using the software program MC Stimulus II (v. 2.4.6).

Begin by connecting the hardware to both a power source and a computer. Setting up dual monitors is advantageous for running the two software programs simultaneously. Ensure that the computer meets the system requirements specified by the necessary software before configuring the software settings required for the experiment. The most critical step is to eliminate background noise such as the 50 Hz main supply frequency. This can be achieved by placing power adapters as remote as possible from the signal-receiving system.

ECG analysis is performed using the ecgAUTO software (v. 2.5.1.17, EMKA Technologies, Paris, France).

For detailed setup instructions and troubleshooting guidance, it is advisable to consult the user manuals provided by the respective software and hardware manufacturers.

### B) Preparation (Timing 15–20 min)

If the experiment is terminal in nature, sterile surgical conditions are not strictly required. However, aseptic conditions are necessary if the experiment is performed with the animal surviving.

1) Prepare the necessary surgical instruments, such as forceps, micro scissors, cannula, and sutures, along with the intracardiac pacing catheter required for catheterization.
2) Apply conductive electrode gel to the defibrillation paddles, turn on the external defibrillator, and select the 5-J energy level.
3) Induce anesthesia by administering a gaseous mixture of 5% isoflurane (Baxter International, Deerfield) in a closed chamber (timing, 1–2 min). Maintain anesthesia using 1.5–2% isoflurane in a 30% O_2_–70% N_2_O mixture via a nose mask.
4) Place the animal in a supine position on a heated ECG-board set at 37°C. Apply conductive electrode gel to electrodes integrated into the board and secure the limbs with tape to enable continuous recording of the ECG.
5) Cover the animal’s eyes with eye ointment to prevent them from drying out.
6) Use a hair clipper to remove fur from the right side of the neck (surgical area) as well as the anterior and posterior thoracic area (areas where electrical shocks might be delivered).
7) Carefully insert a rectal temperature probe to monitor the temperature. Maintain a stable body temperature at 37°C, using an overhead incandescent heating lamp if necessary. It is important to avoid hypothermia as it can cause a progressive decrease in heart rate and increase the risk of arrhythmias (20, 21).

### C) Catheterization of the Right Atrium and Ventricle (Timing 10–20 min)

The cannulation of the right jugular vein follows a well-described method (18, 22).

1) To the right of the midline, create a 1-cm skin incision using forceps and sharp scissors. Then, carefully dissect through the subcutaneous tissue and muscle layer to expose the right jugular vein.
2) Gently isolate the vein from the surrounding tissue. Carefully lift the vein and secure it by placing two 5-0 or 6-0 sutures underneath.
3) Tie a knot at the proximal end of the vein.
4) Form a loose knot with the distal suture and slowly raise the distal end of the vein to straighten it out.
5) Create a small incision in the vein using an angled cannula.
6) Insert a 1.6-Fr octapolar catheter with a 1 mm interelectrode distance (EPR-802, Millar Instruments Inc., Houston) through the incised vein and carefully advance it to the right atrium (RA). Keeping the incision area humid with saline solution can be useful to prevent coagulation, which otherwise can make maneuvering of the catheter demanding.

**Figure 1.**
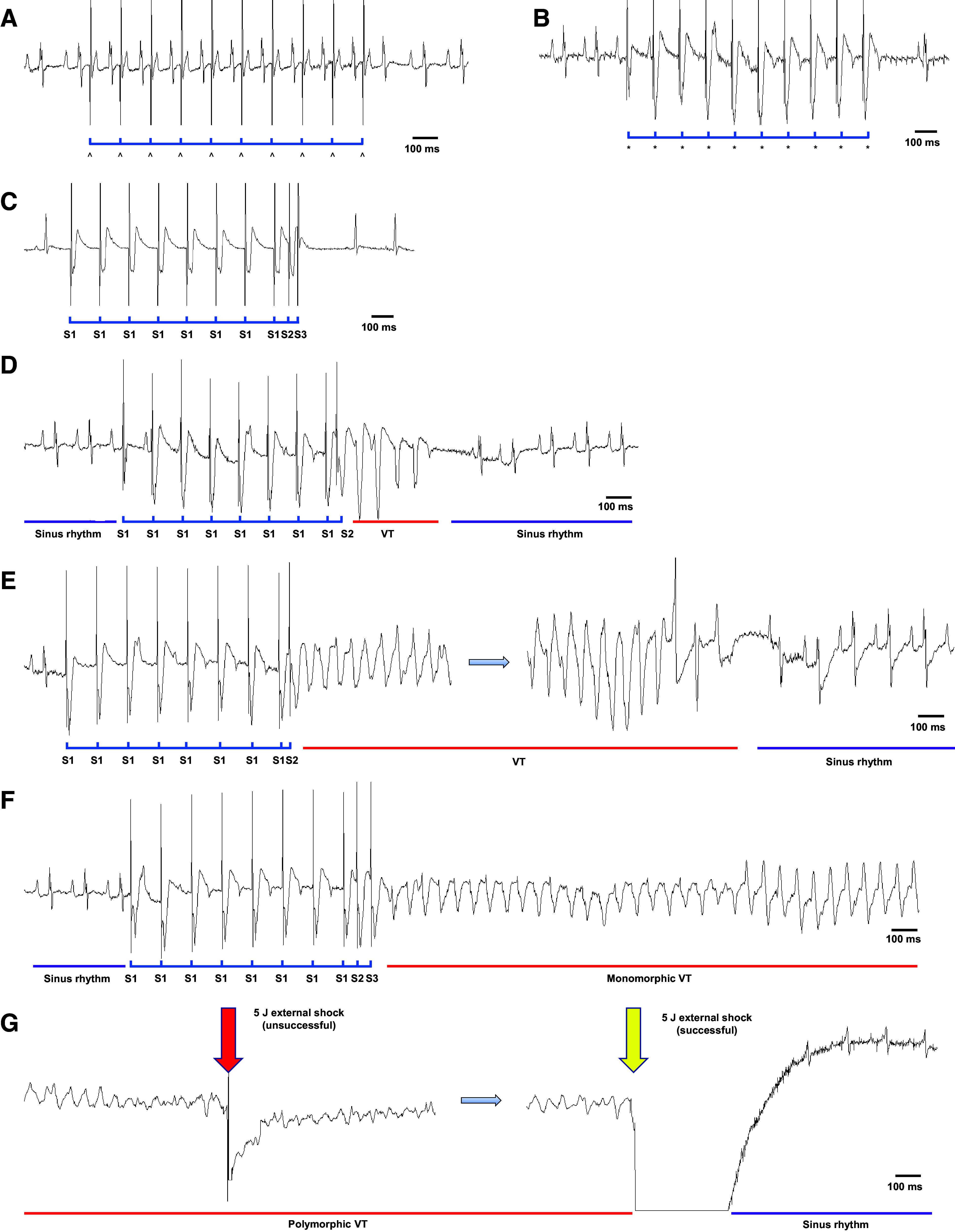
Representative surface ECGs from the in vivo pacing protocol. *A*: determination of the atrial pacing threshold. Each stimulus (^) at a CL of 120 ms results in successful atrial capture. *B*: determination of the ventricular pacing threshold. Each stimulus (*) at a CL of 120 ms results in successful ventricular capture. *C*: ventricular PES (S1–S3) showing successful capture on S1 and S2, but loss of capture on S3. *D*: initial SR followed by ventricular PES (S1–S2) that triggers a short nonsustained VT lasting four beats before returning to SR. *E*: ventricular PES (S1–S2) that induces a nonsustained VT (total duration ∼5.5 s) with spontaneous conversion to SR. *F*: initial SR (with a CL of ∼145 ms) followed by ventricular PES (S1–S3) that induces a sustained VT (with a CL of ∼70 ms) accompanied by a changed axis deviation. *G*: continuation of the arrhythmia shown in *E*, where the monomorphic VT has degenerated into polymorphic VT. A first unsuccessful attempt at external defibrillation with 5 J (red arrow) is followed by a second attempt with 5 J (yellow arrow), which successfully converts the VT back to SR (with a CL of ∼155 ms). Note that the external shock briefly causes deviation of the ECG signal from the baseline. CL, cycle length; ECG, electrocardiogram; PES, programmed electrical stimulation; SR, sinus rhythm; VT, ventricular tachycardia.

Confirmation of atrial placement of the catheter is achieved by observing accurate intracardiac ECG signals and ensuring successful atrial capture (Fig. 1*A*). Advancing the catheter into the right ventricle may pose challenges because of anatomical circumstances. By slightly rotating the catheter counterclockwise while carefully advancing it, a sudden pulsative movement of the catheter may be felt, indicating proximity to the tricuspid valve.

1.  After gentle back-and-forth movement of the catheter, correct placement is verified by observing intracardiac ECG from the right ventricle.
2.  Once the catheter is optimally positioned, secure it by tying a knot on the distal suture to ensure both hemostasis and stable catheter positioning.

### D) Ventricular PES (Timing 10–35 min)

1) The ventricular pacing threshold is determined by applying ten 2-ms current pulses at a cycle length (CL) of 120 ms and with an amplitude of 1,000 mV. With each pacing train, the amplitude is sequentially reduced by 200 mV until the loss of capture. To ensure capture throughout the ventricular stimulation protocol, further pacing is performed at twice the voltage threshold (Fig. 1*B*).
2) The ventricular effective refractory period (VERP) is determined by using a S1–S2 protocol. VERP is defined as the longest S1–S2 coupling interval at which a premature stimulation delivered after the train of pulses fails to result in conduction of the ventricle. After an initial train of eight beats at a fixed CL 120 ms (S1), a coupled premature stimulus (S2) set at 100 ms is applied, and progressively reduced with 5 ms after each pacing train until the loss of capture. When the capture is lost, a new set of protocols are run with the S1–S2 coupling interval starting at 5 ms above the S1–S2 coupling interval at which conduction failed, before being repeated with a 2-ms decrement until the loss of conduction or the initiation of VA.
3) A second extrastimulus (S3) is then introduced. Coupling interval of both S2 and S3 is set at VERP+20 ms. S3 is gradually reduced in 5 ms decrements until the loss of capture (Fig. 1*C*). When the capture is lost, a new stimulus is given with a coupling interval set at 5 ms greater than the S2–S3 coupling interval at which conduction failed, before being repeated with a 2-ms decrement until the loss of conduction. S3 is then set at the shortest coupling interval that preserves S3 capture, before the S1–S2 interval is shortened in same manner until the loss of S2 capture, but with S3 capture still maintained.
4) By introducing a third extrastimulus (S4), the effective refractory period of S3 is determined. Coupling interval of both S2–S3 and S3–S4 is set at VERP+20 ms. The S3–S4 interval is progressively reduced by 5 ms until the loss of capture, then repeated with a coupling interval of 5 ms above the point of lost coupling before being lowered in 2 ms decrements until the loss of capture. S4 is then set at the shortest coupling interval with maintained S4 capture, before S3 and S2 are similarly shortened, respectively.
5) Finally, a fourth extrastimulus (S5) is added to the pulse train with coupling interval S2–S3, S3–S4, and S4–S5 set at VERP + 20 ms. S5 will be reduced until the loss of capture and set at shortest coupling interval with maintained S5. Then S4, S3, and at last S2 are reduced in an analogous way as described in the steps above.

The PES protocol ends when the VERP S1–S5 protocol is completed or if pacing induces sustained VA at any point in the protocol.

### E) Defibrillation (Timing 1–2 min)

It is recommended to use a portable, external defibrillator with manual operating mode. We use a biphasic defibrillator (M4735A Heartstart XL, Philips Medical Systems, Andover) equipped with internal defibrillation paddles. The default shock energy level is set at 5 J.

1) Verify the onset of sustained ventricular tachycardia (VT) by examining the ECG. If interference or artifacts complicates the interpretation of the ECG recordings, observe the breathing pattern of the animal.
2) In the case of VT, wait for the specified time from arrhythmia onset, dependent on the specific study protocol, to determine whether the VT is nonsustained or sustained (Fig. 1, *D*–*F*).
3) Charge the defibrillator and verify the ongoing VA by examining the ECG.
4) Position the paddles over the anterior and posterior thoracic area. For the posterior placement, slide the paddle under the animal and apply slight pressure.
5) Deliver an electrical shock and observe for the return of sinus rhythm and/or breathing (Fig. 1*G*). If VA persists, deliver an additional 5-J electrical shock.
6) If VA persists despite the previous two attempts, increase the defibrillation energy to 7 J and deliver another shock.
7) If the three initial shocks fail to terminate the VA, the decision to administer a repeated shock should be based on the specific study protocol, considering the potential disadvantages associated with multiple shocks, as discussed in *Limitations*.
8) Assess the outcome based on evaluating ECG recordings and conducting clinical assessment, such as monitoring respiratory chest movement.

Be aware that placing the paddles (*step E.5*) and/or delivering electric shocks (*step E.6*) may cause limbs to loosen from the ECG board, causing ECG artifacts.

### F) Excision of the Heart (Timing 1–2 min)

1) Loosen the distal knot, remove the catheter from the jugular vein, and retie the knot again.
2) Although the animal is still under anesthesia, perform a thoracotomy and, with a 30-G needle, inject a bolus of heparin (0.07 mL of a 5,000-IU solution, equivalent to 900–1,000 IU/kg) into the ventricular cavity through the apex to prevent intracardiac thrombosis. Alternatively, consider systemic delivery of heparin intravenously or intraperitoneally.
3) Perform humane euthanasia by following these steps: begin by making an incision starting from under the sternum’s xiphoid process and extend the incision bilaterally to the ends of the costal margins and through the ribs. Gently lift the thorax upward and open the pericardium. Finally, transect the great vessels to remove the heart in accordance with the method previously described (23).
4) Immediately after excision, place the heart in ice-cold Tyrode’s solution, consisting of (in mmol/L) 116 NaCl, 18 NaHCO_3_, 1 MgSO_4_, 5 KCl, 1 Na_2_HPO_4_, 11 glucose, 1.2 Na-pyruvate, and 1.4 CaCl_2_, and bring it to ex vivo experiments with Langendorff perfusion.
5) The ice-cold Tyrode’s solution should be freshly aspirated with a mixture of 95% O_2_–5% CO_2_ at 37°C to provide oxygenation and maintain a pH at 7.4, as previously stated (24).

## EX VIVO PROCEDURES

### G) Langendorff Perfusion and PES (Timing 45–60 min)

Ex vivo PES and ECG recording are performed using Labchart software (ADInstruments, Dunedin, New Zealand). Note that perfusion flow, perfusion pressure, and pacing frequency may vary based on factors such as gender, age, size, and strain of animals.

**Figure 2.**
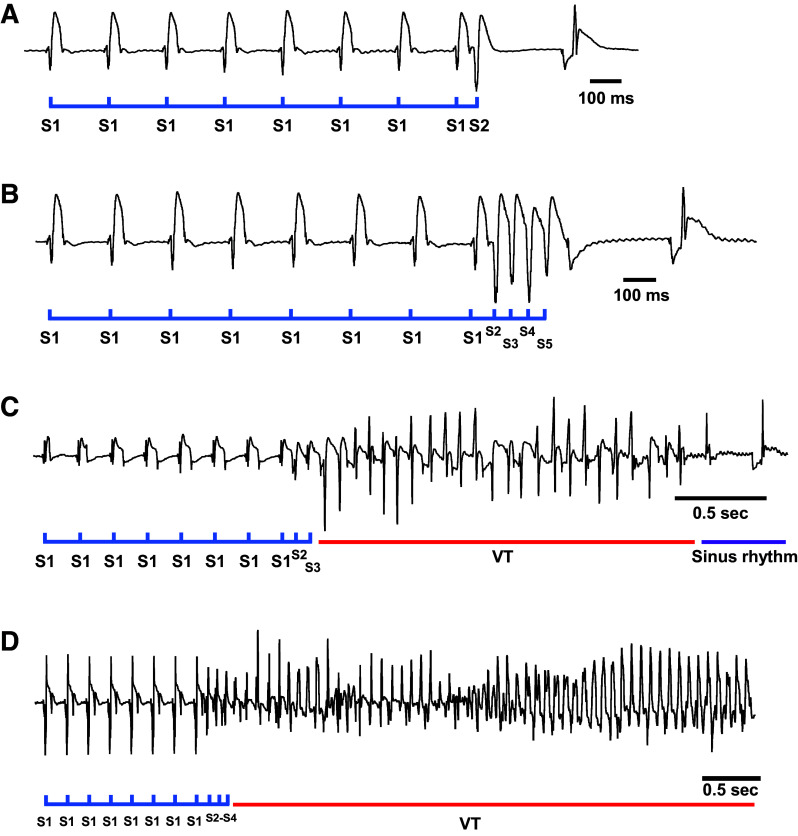
Representative epicardial ECGs from the ex vivo pacing protocol. *A*: example of ventricular PES, S1–S2 in a healthy heart without any arrhythmic behavior. *B*: example ventricular PES, S1–S5 in a healthy heart without arrhythmic behavior. *C*: example of ventricular PES, S1–S3 in a MI heart that triggers a short nonsustained VT, lasting ∼2 s before returning to SR spontaneously. *D*: example of ventricular PES, S1–S4 in a MI heart that induces a sustained VT. ECG, electrocardiogram; MI, myocardial infarction; PES, programmed electrical stimulation; SR, sinus rhythm; VT, ventricular tachycardia.

1) Cannulate the excised heart through the ascending aorta. The maximum perfusion time of a heart preparation is around 3 h. Therefore, the experimental protocol should be planned to conclude within a 3-h timeframe.
2) By using a modified Langendorff working heart setup (Model IH-SR 844, Harvard Apparatus, Holliston), hang the heart vertically and perfuse it retrogradely with oxygenated Tyrode’s solution at 37°C at a flow rate starting at 9 mL/min to maintain a coronary perfusion pressure between 60 mmHg and a maximum of 80 mmHg. Flow rate should be adjusted to sustain the desired perfusion pressure, typically falling within the range of 8–10 mL/min.
3) Monitor the pressure closely with an inline pressure sensor while a temperature probe is lined with the epicardial LV wall.
4) Maintain surface temperature of 37°C by lowering the heart into a water-jacketed glass cell. Place ECG electrodes (Hugo Sachs, Harvard Apparatus, UK) in contact with the left atrium and the LV wall.
5) Perfuse the heart for 30 min to stabilize it before ventricular PES. For a healthy rat heart, expect a stable intrinsic CL between 280 ms and 210 ms (3.5–4.7 Hz).
6) Ventricular PES is achieved by right ventricle endocardial pacing. Make a small incision in the RA before gently inserting the catheter and maneuvering it toward the right ventricle until stable capture is attained.
7) VERP is determined using a S1–S2 protocol in accordance with the in vivo experiments. Evaluation of susceptibility to VA is performed following the in vivo S1–S5 experimental protocol (Fig. 2, *A–D*).

Previous data show that the induction of sustained VA in Langendorff-perfused hearts from sham and MI rats is demanding (25). To challenge the VA susceptibility further, the ex vivo stimulus train protocol could be pursued with a burst pacing protocol.

1.  Ventricular burst pacing can be achieved by right or left ventricle endocardial pacing or LV epicardial pacing by a mini coaxial stimulation electrode (Hugo Sachs, Harvard Apparatus, UK). The amplitude of the burst pacing stimulus, starting at 2 mA, is gradually increased in 1 mA increments every 20 s until either reaching the VA threshold or a defined maximum stimulation current is attained. We suggest a maximum current amplitude of around 30 mA for mice and rats (25).

**Figure 3.**
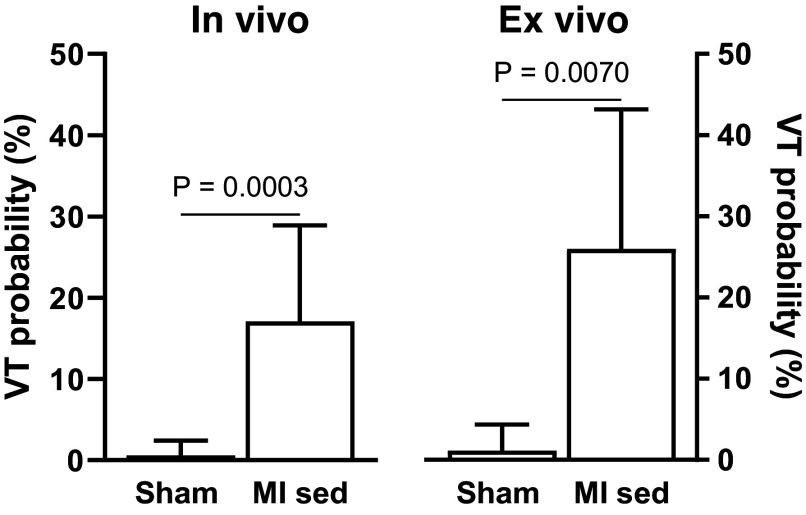
Comparison of VT susceptibility during an S1–S2 protocol in sedentary animals with sham surgery and MI surgery, observed both in vivo and ex vivo. The Mann–Whitney *U* two-tailed test was used to test for group differences. MI sed, sedentary rats with myocardial infarction; VT, ventricular tachycardia.

Figure 3 illustrates mean data on VT susceptibility observed during a S1–S2 protocol. The comparison is between animals with healthy hearts after sham surgery and those with MI. Both groups are studied in vivo and assessed ex vivo using Langendorff perfusion.

### H) Optical Voltage Measurements (Timing 60–90 min)

Following ex vivo PES, prepare the heart for optical measurements of membrane potentials across the epicardial LV wall.

**Figure 4.**
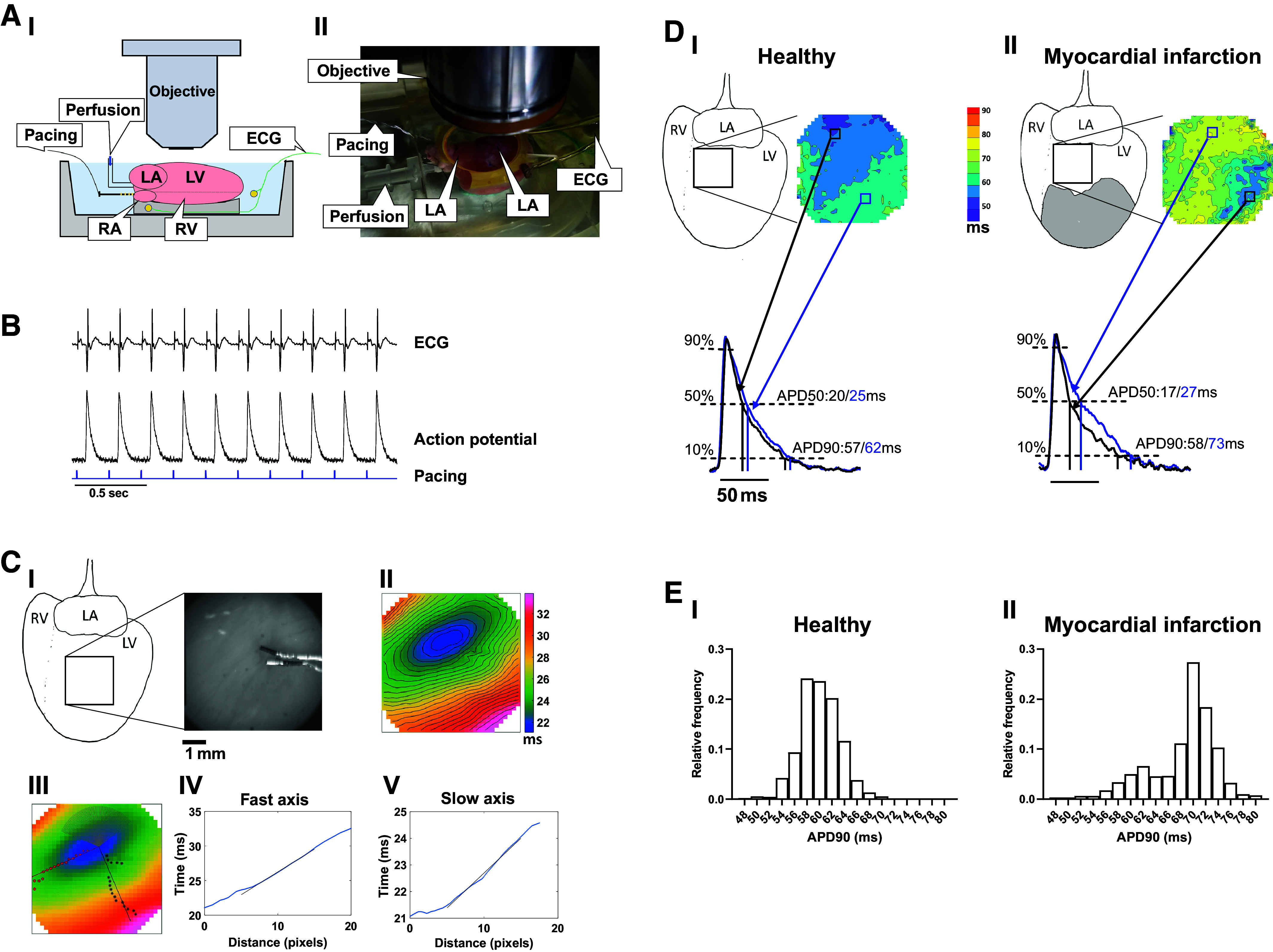
Ex vivo setup and optical mapping. *A*: illustration (I) and a labeled experimental picture (II) of a Langendorff-perfused heart placed horizontally in a temperature-controlled chamber with Tyrode’s solution. ECG electrodes are positioned near the base and apex of the heart, with the pacing electrode on the RA. *B*: simultaneous recordings of ECG and AP from the RA with a 224-ms CL. *C*: the upper panel illustrates the imaging site on the heart (I) with a brightfield image with the pacing electrode, and an isochronal map of AP activation (II). The lower panel shows the fastest conduction axis indicated by red asterisks and slowest conduction axis indicated by black asterisks (III), along with the relationship between time and distance in the fastest axis (IV) and slowest axis (V). *D*: example illustrating regional differences in AP duration (APD_90_ and APD_50_) in a healthy heart (I) compared with a MI heart (II). *E*: pixel frequency distribution of each 2-ms APD_90_ category obtained from mapping of a healthy heart (I) compared with a MI heart (II). AP, action potential; APD; action potential duration; CL, cycle length; ECG, electrocardiogram; LA, left atrium; LV, left ventricle; MI, myocardial infarction; RA, right atrium; RV, right ventricle.

1) To inhibit contraction and minimize movement artifacts, switch the perfusate to a Tyrode’s solution containing 10 mmol/L 2,3-butanedione monoxime (BDM; Sigma-Aldrich, Cat. No. B0753) and 10 µmol/L blebbistatin (Abcam, ab120425). Leave the heart to stabilize for 10 min before loading of the voltage-sensitive dye (VSD) (FluoVolt Dye, PowerLoad Concentrate; F10488, Thermo Fisher, Waltham).
2) Prepare FluoVolt LoadingMix with 20 µL FluoVolt, 200 µL PowerLoad, and 780 µL Tyrode’s solution. Loading of FluoVolt is done through a two-step protocol to ensure high membrane binding affinity of FluoVolt:
  a) Loading: Inject FluoVolt LoadingMix in the perfusion flow through a loading vent right before the cannulated heart, at a flow rate of 1 mL/min.
  b) Loop: Collect the perfusate beneath the heart in a recirculation chamber for 7 min after the start of injection. Use this perfusate in a perfusion loop for 15 min to ensure saturated binding of FluoVolt.
3) While maintaining regular Langendorff perfusion, move the heart carefully from vertical to horizontal position into a temperature-controlled perfusion chamber filled with Tyrode’s solution. Place ECG electrodes near the base and the apex of the heart, respectively.
4) Lay a temperature probe in the bath and place a stimulating electrode according to the chosen protocol (Fig. 4*A*). For this protocol, we used a purpose-built perfusion chamber (Cairn Research, Faversham, UK). These components could also be 3-D printed to meet specific needs. It is essential to consider the chamber’s size, ensuring it is suitable to the species and to prioritize effective and accurate temperature control.
5) Widefield epifluorescence voltage mapping is recorded by a 128 × 128 pixel CardioCMOS-SM128 camera at 5 kHz (RedShirt Imaging; Decatur) via a 2.5×, 0.12 numerical aperture air objective lens with a 505 nm OptoLED excitation source. Emitted light is filtered through an ET535/50 nm (Chroma) bandpass filter.
6) Epicardial voltage maps are obtained from the LV surface. Figure 4*B* displays sample traces of ECG and APs during RA pacing at 4.5 Hz. The pacing site and frequency may vary according to the aim of the protocol.
7) To measure epicardial CV, the electrode should be placed within the field of view before creating isochronal maps. The procedure for CV analysis is outlined in “I) Optical mapping analysis” (*step I.2*). During normal conduction (i.e., during RA pacing), the spatial distribution of the AP duration (APD) and AP upstroke can be derived (*steps I.3* and *I.4*). In Fig. 4*D*, I, an isochronal map of APD_90_ from a typical healthy heart is displayed, alongside AP traces from the region with the shortest (black) and longest (blue) AP. Figure 4*D*, II presents the corresponding data from a typical MI heart. In Fig. 4*E*, I and II, the frequency distribution of APD_90_ from the healthy heart and the MI heart is respectively illustrated.

To obtain further mechanistic insights, various pacing protocols and/or ion-channel inhibitors, or other chemical agonists/antagonists may be used. Choice of protocol depends on the specific aim of the study. High-frequency pacing or S1–S2 extrastimulus protocols have the potential to reveal asymptomatic electrophysiological defects.

**Figure 5.**
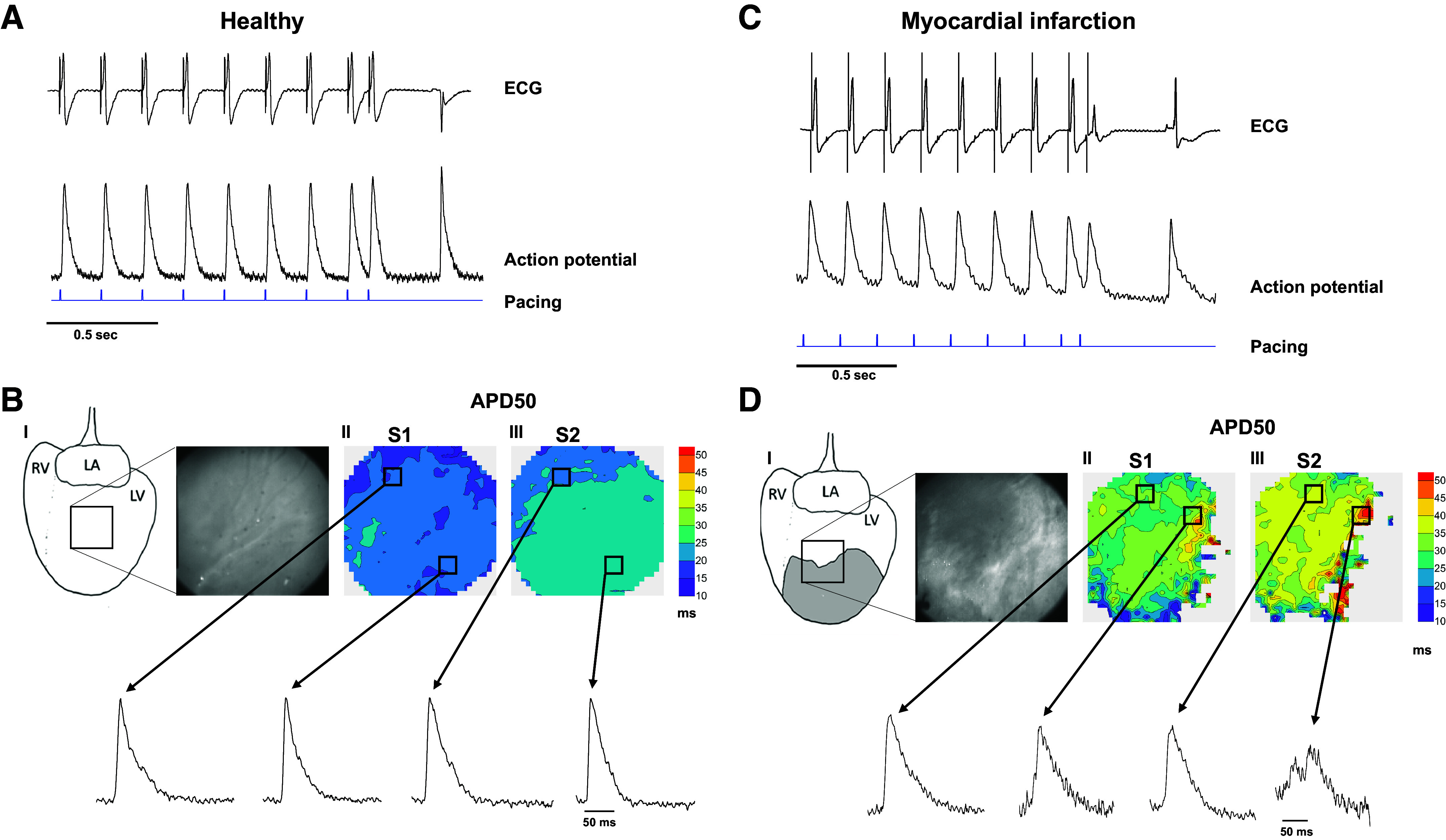
Example of optical mapping during S1–S2 protocol in healthy and MI hearts. *A*: ECG (*upper panel*) and spatially averaged AP (*lower panel*) from a healthy heart during ventricular S1–S2. *B*: the *upper panels* illustrate site of imaging (I) with a brightfield image of the epicardial surface and isochronal maps of APD_50_ comparing S1 (II) and S2 (III). The *lower panels* show APs from different regions with little variation between S1 (arrows on the far *left*) and S2 (arrows on the far *right*) in a healthy heart. *C*: ECG (*upper panel*) and spatially averaged AP (*lower panel*) from a MI heart during the ventricular S1–S2 protocol. *D*: the *upper panels* illustrate site of imaging (I) with a brightfield image of the epicardial surface where the white parts correspond to connective tissue/infarction. II is an isochronal map of APD_50_ during S1, and III shows the corresponding isochronal map of S2. The *lower panels* show that APs from different regions during S1 deviate somewhat (arrows on the far *left*), but with the addition of S2, the AP from the border zone of the MI (arrows on the far *right*) exhibits substantial alterations compared with the AP from S1. AP, action potential; APD; action potential duration; ECG, electrocardiogram; LA, left atrium; LV, left ventricle MI, myocardial infarction; RA, right atrium.

Figure 5*A* shows ECG (*upper panel*) and APs (*lower panel*) from a spatially averaged LV area in a healthy heart (Fig. 5*B*) during a S1–S2 protocol. Figure 5*B* also shows the isochronal maps from S1 to S2 with the corresponding AP traces from the same small regions during S1 and S2. Figure 5*C* shows ECG (*upper panel*) and the spatially averaged APs (*lower panel*) from the border area in an MI heart (Fig. 5*D*). The ECG shows a delayed and lower amplitude signal in S2 (Fig. 5*D*) compared with S1 (Fig. 5*C*). Looking at different regions, AP generation starts to fail in the transition from the noninfarcted area to the infarcted area (Fig. 5*D*, the box furthest to the right in the isochronal image of S1 and S2, respectively).

### I) Optical Mapping Analysis

Widefield optical mapping data are analyzed using Optiq AP analysis software (Cairn Research, Faversham, UK), and a custom script written in MATLAB (MathWorks, Natick) is used to calculate CV. Below, we will briefly discuss CV and AP measurements, with more comprehensive details in Laughner et al. (26).

1) Numerous factors can influence signal quality, and each setup will have its limitations. Besides using hardware designed for the task, we emphasize the crucial role of the loading procedure, as discussed in “H) Optical voltage measurements.” To improve signal-to-noise ratio, pixels can be binned, and filters applied to remove excess noise. However, this may reduce spatial resolution. Temporal averaging of the voltage signal will increase the signal-to-noise ratio and often reduce the need for filtering. To ensure correct averaging, we recommend using the stimulus pulse as the marker for each AP. The ECG could also be used to synchronize the APs for averaging. In the current analysis, we used 4 × 4 binning in addition to a Gaussian temporal filter and averaging where suitable. Although binning reduces spatial resolution, it retains temporal information, which is important for determining AP upstroke and measuring CV. Filters should always be used cautiously to avoid overfiltering and must be adopted to suit the specific purpose and system. For an extensive review of filter options, please refer to Laughner et al. (26).
2) Epicardial pacing (in the field of view) can be used to derive CV in epicardial tissue (Fig. 4*C*, I and II). CV was calculated as follows:

Initially, we located the pixel (T0) on the activation time map where the earliest activation occurred. This minimum time value was denoted as T0. Following this, a fixed range of angles (120°) around T0 was excluded, especially when T0 was positioned near the map’s edge or when the map contained pixels lacking valid activation time values. We then scanned circular arcs within the remaining 240° circular sector centered at T0, covering a set range of radial distances (5–15 pixels) from T0, to locate the points indicating the earliest and latest time in relation to T0. The median angles of the earliest and latest points were considered as the longitudinal and transverse axes, respectively (Fig. 4*C*, III). These axes were perpendicular in all cases, as would be expected from typical epicardial activations. Finally, the slopes of the time versus distance profile lines between 5 and 15 pixels from T0 were estimated for both axes via robust linear regression in MATLAB’s “fitlm” function using the “RobustOps” option. These values were then expressed as CV in cm/s (Fig. 4*C*, IV and V). This entire process was repeated for each activation map, and the resulting plots were examined to ensure the credibility of the angles and profiles found. We recommend using 50% depolarization as the activation time because it is less susceptible to the effects of signal noise. In the provided example, the CV was 72 cm/s in the fastest direction and 29 cm/s in the slowest direction.

1) Defining the start of the AP upstroke as the time when fluorescence reaches 50% of peak amplitude, with interpolation between sample points, is preferred because accurately determining the time of maximum upstroke velocity (dV/dtmax) from optical data can be challenging (27). The AP rise time is measured as 10–90% of the upstroke. APD can be measured from 50% depolarization or peak AP amplitude to 50, 75, and/or 90% repolarization (Fig. 4*D*). Another measure proven to predict arrhythmic behavior is the ratio of 30% (or 50%) and 90% repolarization times, often referred to as “triangulation.”
2) Dispersion in activation and repolarization is strongly associated with an increased risk of VA and are typical manifestations of cardiovascular disease (15). Various methods can be used to calculate and visualize this heterogeneity. Isochronal maps offer descriptive visuals (Fig. 4*D*). Frequency distributions can be plotted to illustrate the distribution of the data (Fig. 4*E*). The simplest method for quantifying data spread globally is the standard deviation. However, we recommend using the mean interquartile range (IQR), which measures the spread of 50% of the data around the mean, covering the first to third quartile. Likewise, the spread of data from the 5th percentile to the 95th percentile (IP90) has shown to predict an increased susceptibility to arrhythmias. In pathological conditions, the distribution of EP data can become skewed. It is advisable to present the data as a range, either within the 50th or 90th percentile, in addition to the median. Lammers and coworkers developed a local inhomogeneity index (28). In this approach, each of the 2 × 2 pixel quadrants is assigned a number representing the largest difference in activation time between the pixels, referred to as the phase difference. Subsequently, all the phase difference numbers are sorted, and the range between 5th and 95th percentiles is divided by the median. This calculation assigns a relative local inhomogeneity value to each heart.
3) Beat-to-beat variation or alternans can be assessed by analyzing AP parameters recorded individually for each AP separately. Where 2:1 alternans has been identified, it is possible to average every other AP to increase the signal-to-noise ratio. Higher pacing frequencies reduce the safety factor for conduction. Therefore, all mentioned parameters should include higher pacing frequencies. An S1–S2 pacing protocol provides information about sudden shortening of coupling interval, enabling the determination of pathological conduction of the AP even when a low pacing frequency cannot observe the pathological AP waveform.

## DISCUSSION

VAs remain a potential life-threatening complication of coronary artery disease and heart failure. Greater understanding of arrhythmogenesis and advances in technology have led to modern long-term therapies, which include pharmacotherapy with antiarrhythmic drugs, catheter ablation, and device therapy with implantable cardioverter defibrillators (29, 30).

In this study, we have presented a comprehensive protocol for an in vivo and ex vivo EP analysis with optical mapping in an arrhythmogenic rat model. This protocol can be flexibly used to investigate underlying mechanisms of VA and to evaluate effects and mechanisms of different antiarrhythmic interventions.

### Advantages and Possibilities of the Model

The advantage of a study that combines both in vivo and ex vivo methods in the same heart is the opportunity to support or challenge the findings of each method using results obtained from the other. This allows us to track the ex vivo findings with in vivo behavior, where physiological processes and disease features remain unaltered. Another strength of this protocol lies in combining the benefits of more physiologically accurate conditions in vivo with the controlled and comprehensive measurement capabilities in the ex vivo setting. Using identical pacing protocols in vivo and ex vivo, we can support that the clinically relevant pathophysiological features are conserved in the ex vivo model, where manipulation of perfusion, agonists, and antagonists is better controlled and high-resolution EP measurements like optical mapping can be used.

Defibrillation of sustained VA allows the possibility for multiple cycles of EP measurements or other experiments, depending on each specific protocol (31, 32). Long-lasting VA may affect further experiments, as sustained arrhythmias may prompt metabolic alteration of the myocardium (33).

Optical mapping adds valuable information of the AP waveform shape across the epicardial surface. This technique enables the characterization of CV, AP activation, and duration.

In addition, it can provide information about the dispersion of AP activation and duration. These measurements can use the corresponding widefield image as a reference to distinguish the scar tissue, border zone, and remote nonischemic myocardium.

Standard optical mapping requires the use of mechanical uncouplers to minimize movement artifacts. However, these mechanical uncouplers may influence the electrophysiological properties, necessitating ex vivo PES procedures to assess the proarrhythmic status before imaging. We used a combination of two chemically distinct mechanical uncouplers: butadiene monoxime (BDM, 5 mM) and blebbistatin (10 µM) to reduce mechanical activity, in line with previous studies (24, 27, 34, 35). Other studies have shown that, at these concentrations, these mechanical uncouplers have minimal effects on cardiac electrophysiology (35–39). Blebbistatin acts by specifically inhibiting myosin at the site of interaction with the actin myofilament (40). BDM has multiple sites of action, affecting both myosin and actin (38). The combined actions through independent pathways minimize movement artifacts during single-wavelength voltage measurements in cardiac muscle. Blebbistatin alone would be sufficient in reducing movement artifacts during lower-resolution recording as described in the current paper. However, when single-cell resolution is required, it is advisable to combine blebbistatin and BDM.

The proposed PES protocol is based on standard protocols used in clinical practice, making the experimental results more applicable and translational to human cardiology.

Alternative approaches, such as burst pacing, stimulation using more complex pacing protocols, or the use of pharmacological agents (e.g., epinephrine or caffeine), are also viable options.

This protocol can be used for a systematic evaluation of established models of cardiac disease or in new models, including genetic variants with uncertain risk of VA. It can also be used for evaluation of antiarrhythmic interventions, such as novel antiarrhythmic drugs and/or lifestyle interventions like exercise and nutritional changes.

Although the primary focus of this protocol is ventricular arrhythmogenesis, its underlying principles can also be extended and applied to the study of atrial arrhythmias (41).

### Practical Advice and Possible Challenges

#### In vivo.

- Accurate setup of the acquisition hardware is critical to obtain acceptable ECG signals and avoid artifacts (42). It can be challenging to avoid signal noise and troubleshooting may be needed. In our set up, the 50-Hz noise is avoided by placing the power adapter from the various electrical devices at a sufficient distance from the ECG board.
- Correct placement of the catheter into the right ventricle for satisfactory capture may be challenging. In case of resistance when advancing the catheter through the tricuspid valve, simultaneously try *1*) a slight twirling of the catheter or *2*) a careful shifting of animal position to one side or by lightly elevating the animal by placing a few fingers underneath.
- If an acceptable ECG signal is lost during positioning, the catheter could be misplaced in the inferior vena cava.
- In post-MI animals with cardiac remodeling, there might be adequate ECG signals during positioning, but still unsatisfactory capture during pacing. This can be solved by a minimal adjustment of catheter placement.

#### Ex vivo.

- The time from excision of the heart until cannulation and stable retrograde perfusion pressure is established, is critical to avoid ischemia. Maintaining a stable physiological epicardial surface temperature is also crucial to avoid temperature-induced arrhythmias.
- Total duration of PES and optical mapping protocols should be carefully monitored, as the Langendorff heart will deteriorate and eventually influence the quality of EP and conduction system. In these studies, a maximum time of 3 h for the ex vivo phase of the experiments were used.
- PES recordings should be performed ahead of optical mapping because of EP effects induced by electromechanical uncouplers like BDM and blebbistatin. This step is necessary to avoid optical movement artifacts.
- FluoVolt is an expensive VSD. However, it is a novel fast-response VSD with lower toxicity and higher dynamic range than traditional VSDs (24). Adequate loading of FluoVolt is determined by concentration, flow rate, and time of exposure. To lower the concentration of FluoVolt, the time of exposure is increased through a looping and recirculation of the perfusate. Recirculation of the perfusate is time limited as the procedure might impair the cardiac function, thus this step must be carefully monitored.
- Concentration of FluoVolt is also dependent on heart size. The looping and recirculation protocol is not necessary for mice hearts. However, if larger hearts (e.g., rabbit) are of interest, time should be spent on optimizing the loading protocol.

### Limitations

Although small and large animal models have provided significant knowledge of arrhythmia mechanisms, there obviously remains limitations in translating findings from a specific animal model to the heterogeneities in human cardiac disease (43, 44). Notably, there are differences in EP properties in rats and humans (45). The main consideration of this model is that VA is induced by EP pacing, as the mechanisms behind spontaneously occurring VA might be different. However, a study based on spontaneous nonsustained VT would be challenging as these arrhythmias are rare in most animal models (46, 47). Also, the MI is invoked by ligation of a pivotal coronary artery in an otherwise healthy heart.

In our ex vivo model, the vagus nerve is not preserved, leading to alterations in autonomic nervous system activity. These changes may have had an impact on arrhythmogenesis in vivo. Previously, a model for heart preparation, which maintains intact innervation by the vagus nerve, has been described (18).

Isoflurane is a commonly used inhalant anesthetic in small animal experiments due to its ease of control over both the duration and depth of anesthesia. Although it may potentially affect cardiac function and electrophysiological metrics, its cardiac effects are generally less disadvantageous than those of other anesthetics (48, 49).

The electrical energy delivered during defibrillation could potentially alter diastolic function, reduce myocyte contractility, and change intracellular calcium dynamics (50). Restoring sinus rhythm by external defibrillation could therefore in theory have had an influence on the subsequent ex vivo experiments. There is a knowledge gap concerning the maximum number of electrical shocks that can be administered before initiating ex vivo studies. Some resuscitation studies have examined the delivery of up to three shocks before resuming precordial compression. In our study, we successfully terminated VAs and restored sinus rhythm using no more than three shocks, most often fewer.

The main limitations in optical mapping are the transmural resolution and the alterations in electrophysiological properties induced by mechanical uncouplers. The epicardial fluorescence signal is obtained from a volume of a few hundred cubic microns of the epicardium. To obtain cellular resolution in the transmural axis, a two-photon microscopy approach is required and could be added to the proposed protocol (27, 35). Applying mechanical uncouplers like BDM and blebbistatin is preferred to reduce motion artifacts that can influence the optical signal obtained by a camera. However, caution is warranted when interpreting the electrophysiological recordings (27).

### Conclusion

The presented protocol can serve as a valuable tool for investigating novel pathways to prevent or mitigate VA. By trying to integrate the knowledge of local cellular pathology into the mechanisms of in vivo VA, this protocol could offer insights that can contribute to the development of effective interventions. The protocol is suitable for studying various factors such as pharmacotherapy, exercise, diet, radiation, ablation, or genetic modification. Moreover, the applicability of this protocol extends beyond rodents, potentially enabling its use in different disease models and other animal species, including both females and males.

Overall, this protocol holds promise for advancing our understanding of VA and facilitating the development of targeted interventions in cardiac research.

## DATA AVAILABILITY

Data will be made available upon reasonable request.

## GRANTS

This work was supported by Research Council of Norway Grant 241040, Joint Research Committee between St. Olavs hospital and the Faculty of Medicine and Health Sciences, NTNU, Grant 7/38297-99, and Liaison Committee for education, research, and innovation in Central Norway Grant 2017/38202.

## DISCLOSURES

No conflicts of interest, financial or otherwise, are declared by the authors.

## AUTHOR CONTRIBUTIONS

T.O.S., A.B.J., G.L.S., and J.P.L. conceived and designed research; M.N., T.O.S., A.B.J., and K.G. performed experiments; M.N., T.O.S., A.B.J., K.G., F.L.B., G.L.S., and J.P.L. analyzed data; M.N., T.O.S., A.B.J., K.G., F.L.B., G.L.S., and J.P.L. interpreted results of experiments; M.N., T.O.S., A.B.J., and K.G. prepared figures; M.N., T.O.S., A.B.J., K.G., G.L.S., and J.P.L. drafted manuscript; M.N., T.O.S., A.B.J., K.G., F.L.B., G.L.S., and J.P.L. edited and revised manuscript; M.N., T.O.S., A.B.J., K.G., F.L.B., G.L.S., and J.P.L. approved final version of manuscript.
